# Long-term familial Mediterranean fever remission on successful hepatitis C virus treatment in a patient not responding to colchicine: a case report

**DOI:** 10.1186/s13256-018-1691-2

**Published:** 2018-05-18

**Authors:** Manik Gemilyan, Gagik Hakobyan, Susanna Ananyan

**Affiliations:** 1 Department of Internal Medicine, Yerevan State Medical University, 2 Koryun st, 0025 Yerevan, Armenia; 2 Department of Gastroenterology, “Armenia” Republican Medical Center, Yerevan, Armenia

**Keywords:** Familial Mediterranean fever, Chronic hepatitis C, Interferon, Inflammasome, Autoinflammatory disorders, Colchicine resistance, Case report

## Abstract

**Background:**

Familial Mediterranean fever is an autosomal recessive disorder characterized by periodic febrile attacks of aseptic serositis and/or arthritis. The main treatment is colchicine which prevents attacks in the majority of patients except for a group of colchicine-resistant cases. Chronic hepatitis C is a viral infection causing chronic inflammation of liver tissue (hepatitis) which ultimately progresses to fibrosis and liver cirrhosis with a high chance of hepatocellular carcinoma. However, we found no data in the literature concerning the impact of hepatitis C on the course of attacks of familial Mediterranean fever.

**Case presentation:**

We report a case of a 21-year-old white woman with familial Mediterranean fever who had not been responding to a high dose of colchicine (2 mg/day). She presented to our clinic with a finding of chronic hepatitis C genotype 3 infection. After successful antiviral therapy with peginterferon and ribavirin, she became attack-free for 2 years and went on to a lower dose of colchicine.

**Conclusions:**

This unusual case illustrates complete resolution of attacks of autoinflammatory disease after drug-induced clearance of chronic hepatitis C infection. Coexisting infections should be viewed as potentially altering the course of autoinflammatory disorders, and any attempt to cure the infections should be made in order to gain an added value of benefiting the chronic disease. This case highlights the interrelation of external pathogen-related and genetically inherited alterations in immunity and the importance of considering the whole spectrum of possible causative factors rather than implementing separate guidelines in order to achieve best quality of medical care in any given patient.

## Background

Familial Mediterranean fever (FMF) is a genetic, autosomal recessive disorder affecting nations historically inhabiting the Mediterranean basin, including Jews, Armenians, Arabs, Turks, and others. It clinically manifests as periodic, spontaneously occurring and spontaneously resolving febrile attacks of aseptic serositis (pleuritic or peritonitis) and/or arthritis. The major complication is systemic amyloid A (AA) amyloidosis due to deposition of serum amyloid A (SAA) which predominantly affects kidneys and leads to nephrotic syndrome and renal insufficiency [1].

The etiology of the disease is a missense mutation in the Mediterranean fever gene (*MEFV*). The gene is located on the short arm of the 16th chromosome and encodes the synthesis of a protein called pyrin or marenostrin. The biological function of pyrin is not fully elucidated; however, it is suggested to include sensing of intracellular signals directed toward activation of the inflammasome [2]. Inflammasome is a complex molecular assembly in innate immune cells that is activated by different pathogen-associated and damage-associated molecular signals leading to inflammatory response. Among the best described types is NOD-like receptor family, pyrin domain containing 3 (NLRP3) inflammasome, the activation of which promotes a cascade of molecular reactions ultimately resulting in production of activated interleukin (IL)-1-beta and IL-18 [3]. Several genetic mutations cause different types of inflammasome dysfunction forming a group of hereditary autoinflammatory diseases, with FMF being a prototypic disorder of this kind [4]. As a result of *MEFV* mutation, defective forms of pyrin are present and abnormally heightened innate immune response with inflammatory bouts occurs [5].

The universally accepted efficient method of FMF treatment is the lifelong administration of colchicine which prevents inflammatory attacks of the disease as well as its main complication: systemic AA amyloidosis. Colchicine dosage in adults varies from 1 mg/day to the maximum of 3 mg/day, and some 10% of patients still experience attacks despite the highest dosage of the drug [6, 7]. These patients constitute the challenging group of colchicine-resistant cases, and a few alternative therapeutic options have been explored with varied efficiency, including IL-1 directed therapy, anti- tumor necrosis factor (TNF)-alpha compounds, and others [8].

Chronic hepatitis C is a viral infection causing chronic inflammation of liver tissue (hepatitis) which ultimately progresses to fibrosis and liver cirrhosis with a high chance of hepatocellular carcinoma. Until the last decade or so, the main treatments of chronic hepatitis C virus (HCV) infection were double or triple therapy schemes based on pegylated interferon (IFN) alpha. IFN alpha is a molecule normally produced by virally infected cells in the body to render the other cells resistant to viruses. As a drug, it is considered an immunomodulator which stimulates host’s immunity to combat HCV infection. The endpoint of antiviral treatment of HCV is cure which is estimated by sustained virological response (SVR) defined as undetectable viral levels immediately and 6 months after completing therapy. The rates of SVR with peginterferon+ribavirin therapy have been variable depending on virus genotype and different host factors, comprising approximately 70–80% in genotype 3 HCV infection [9].

Both described conditions involve activation of innate immune response mechanisms, and it would be plausible to predict certain interactions between pathological pathways if they are present together in the same patient. However, we have not identified any published data on patients who concomitantly have FMF and hepatitis C, regarding alterations of the natural course of either of the two diseases in the presence of the other, as well as changes in treatment response to FMF with cure of HCV. Our case is therefore a valuable addition to our knowledge of immune mechanisms as an example of how a chronic viral infection and its cure may interact with immunity and the natural course of another immune-mediated disorder in a patient with both conditions.

## Case presentation

We present a case of a 21-year-old white woman who presented to our clinic in September 2013 complaining of weakness and fatigue. She had a history of FMF diagnosed at childhood. Genetic study had revealed homozygous mutation (type M694 V) of *MEFV* gene. She was taking colchicine 2 mg daily but still experiencing attacks.

Her family history was unremarkable; she was a student at university, she had no illicit drug history, and she was not taking medications other than colchicine. She did not smoke tobacco, she was a social drinker, and had a monogamous relationship with a male partner who was HCV-negative. We were not able to identify the source of HCV infection in this patient despite meticulous history taking.

A physical examination revealed blood pressure 100/70 mm Hg, pulse 68 beats per minute (BPM), body temperature 36.6 °C, and no organomegaly on abdominal palpation. No abnormalities were detected on neurological examination. Laboratory findings included alanine aminotransferase (ALT) 52 U/L, aspartate aminotransferase (AST) 40 U/L, and normal gamma-glutamyltransferase (GGT), alkaline phosphatase (ALP), bilirubin, creatinine, albumin, prothrombin, and positive HCV antibodies. An ultrasound detected splenomegaly (15.6 cm in length) and no hepatomegaly. Upper gastrointestinal (GI) endoscopy revealed stomach ulcer, *Helicobacter pylori* (*H. pylori*) positive, and no varices. Eradication therapy was started with first-line triple therapy (omeprazole, amoxicillin, clarithromycin for 10 days), which failed to achieve successful elimination of *H. pylori*, so we went on to prescribe second-line quadruple bismuth-based scheme (omeprazole, bismuth subcitrate, tetracycline, metronidazole for 14 days).

We have previously reported an effect of successful *H. pylori* eradication on attack frequency and severity in a series of colchicine-resistant cases [10]. In this patient, the first attempt of anti-*Helicobacter* therapy did not produce any significant effect on attack pattern; however, she did report some attenuation of attack severity while on second-line treatment which led to successful eradication. This effect occurred without increasing the dosage of colchicine at that time; however, her attacks did not resolve completely.

The decision to start anti-HCV therapy was made based on the following considerations. Our patient had no fibrosis (F0) on shear wave elastography; she had genotype 3 HCV infection with viral load of 1.3 million IU/mL. She was started on pegylated IFN alpha-2a 180 mcg/week and ribavirin 800 mg/day, continuing intake of colchicine 2 mg/day. The duration of antiviral therapy was 24 weeks.

Over the course of treatment, drug side effects were minimal: she did not report flu-like symptoms or fever, and the extent of neutropenia and thrombocytopenia that occurred during treatment did not require dose reduction or withdrawal of medications. Following the protocol of response-guided treatment, HCV RNA counts were evaluated on weeks 4, 8, and 12 and at the end of the treatment (24 weeks), with undetectable viral load at end-of-treatment. She further showed 24-week and 48-week SVR, and virological cure was documented.

The remarkable finding was that during the whole course of treatment and subsequent follow-up (which now has comprised 3 years after the end of therapy) she remained FMF attack-free while taking colchicine in the same dosage as she did when presenting to our clinic and reporting attacks at a frequency of approximately 1 to 2 per month. The laboratory markers of inflammation, which were white blood cells (WBC) count, erythrocyte sedimentation rate (ESR), and CRP, have also remained normal throughout the course of follow-up (see Table 1 for timeline).

**Table 1:** Timeline of the patient

Date	Event
2007	Diagnosed with FMF, started taking colchicine
2007–2014	Taking colchicine 2 mg/day, attacks continued
2013 September	Diagnosed with chronic HCV infection, genotype 3, F0, and *Helicobacter pylori* infection
2013 September to 2014 January	Received two subsequent courses of *Helicobacter pylori* eradication; eradication confirmed. Reported some attenuation but not resolution of attacks
2014 March to August	Received 24 weeks of treatment with interferon+ribavirin, continued colchicine. Reported resolution of FMF attacks
2014 March to 2015 August	Complete resolution of FMF attacks while on same colchicine therapy
2015 August	48-week SVR documented (HCV virological cure)
2015 August to 2017 August	Complete resolution of FMF attacks and normalization of laboratory markers on inflammation while on same colchicine therapy

*FMF* familial Mediterranean fever, *HCV* hepatitis C virus, *SVR* sustained virological response

## Discussion

This case demonstrates how successful eradication of bacterial and viral infections in a patient with colchicine-resistant FMF has remarkably improved colchicine response, possibly due to beneficial effects on innate immunity which is involved in all coexistent disorders of our patient. This is, to the best of our knowledge, the first case report that shows an association between successfully treated chronic HCV infection and colchicine response to FMF.

In order to explain the restoration of response to colchicine after elimination of HCV in this patient who had originally not responded to 2 mg/day dosage, we must look into potential mechanisms of interaction between IFN and colchicine and IFN-induced alterations of inflammatory reactions, particularly in the setting of FMF. Type I IFN has been shown *in vivo* to reduce lipopolysaccharide (LPS)-stimulated levels of IL-1 beta in whole blood but only when added before LPS induction and not concomitantly or afterwards [11]. Other studies have also shown decreased IL-1 and inflammasome activity by type I IFN [12], and mechanisms of this interaction and potential therapeutic uses of IFN in inflammatory disorders including FMF have been nicely summarized in a review [13].

There have been several reports of the effect of IFN on FMF, particularly in colchicine-resistant patients. One showed improvement of attacks in a patient receiving IFN for hepatitis B and re-introduction after cessation of IFN [14], another report showed improvement of knee arthritis over a 3-month IFN treatment course [15]. Two studies involving seven to ten patients reported a beneficial effect of IFN when administered during the attacks [16, 17], and another study reported a beneficial effect of continuous administration in a group of eight patients [18]. In a placebo-controlled study Tunca *et al.* randomly treated 34 episodes of attacks with either placebo or IFN 5 million IU, and found no significant difference in duration of attacks or levels of several inflammatory markers in serum between the two groups [19]. In summary, the beneficial effect of either short-term or long-term IFN co-administration with colchicine on FMF attacks in the setting of no or partial clinical response to colchicine has not been convincingly established.

The interaction between IFN and colchicine has not been fully studied. Although colchicine has been suggested as a potential anti-fibrotic medication, one study was published on patients with HCV treated with conventional IFN that compared viral clearance between groups receiving IFN only and IFN plus colchicine, and the results indicated that cure rates were significantly lower in the latter group [20].

Regarding our patient’s data in light of published literature, this long-term resolution of FMF attacks and inflammation is not likely to be attributable to IFN, since the complete disappearance of attacks was sustained well after discontinuation of antiviral therapy, which is not consistent with the evidence reviewed above. Therefore, this effect could be attributed to elimination of chronic HCV infection which was probably triggering an even more intensified innate inflammatory response in our patient who had an already lowered threshold for inflammation caused by her FMF genotype.

Although innate immunity evolved much earlier in evolution, it received scientific attention much later compared to the adaptive immune system, and so is the case with chronic viral hepatitis. Research has focused on the interference of HCV with the host’s innate immune system over the past decades, and it has shown that HCV has the effect of activating NLRP3 inflammasome to induce IL-1 and IL-18 production [21–23]. It therefore appears plausible that eradication of the virus in a patient with defective NLRP3 inflammasome function could lower the inflammatory burden and allow for better therapeutic effect of colchicine.

## Conclusion

We described an unusual case which illustrates complete resolution of attacks of autoinflammatory disease after drug-induced clearance of chronic hepatitis C infection. It can be learned from our case that any coexisting infections including *H. pylori* and HCV should be viewed as potentially altering the course of autoinflammatory disorders such as FMF, and any attempt to cure the infections should be made in order to gain an added value of benefiting the chronic disease. This case can be viewed as yet another confirmation of the interrelation of external pathogen-related and genetically inherited alterations in immunity and the importance of considering the whole spectrum of possible causative factors rather than implementing separate guidelines in order to achieve best quality of medical care in any given patient.

## Abbreviations

AA: Amyloid A
ALP: Alkaline phosphatase
ALT: Alanine aminotransferase
AST: Aspartate aminotransferase
BPM: Beats per minute
ESR: Erythrocyte sedimentation rate
FMF: Familial Mediterranean fever
GGT: Gamma-glutamyltransferase
GI: Gastrointestinal
*H. pylori*: *Helicobacter pylori*
HCV: Hepatitis C virus
IFN: Interferon
IL: Interleukin
LPS: Lipopolysaccharide
NLRP3: NOD-like receptor family, pyrin domain containing 3
SAA: Serum amyloid A
SVR: Sustained virological response
TNF: Tumor necrosis factor
WBC: White blood cells
